# Correction: Novel diagnostic potential of miR-1 in patients with acute heart failure

**DOI:** 10.1371/journal.pone.0320341

**Published:** 2025-03-10

**Authors:** Seyyed-Reza Sadat-Ebrahimi, Aysa Rezabakhsh, Naser Aslanabadi, Milad Asadi, Venus Zafari, Dariush Shanebandi, Habib Zarredar, Elgar Enamzadeh, Hamed Taghizadeh, Reza Badalzadeh

There are errors in the author affiliations. The correct affiliations are as follows:

Seyyed-Reza Sadat-Ebrahimi^1^, Aysa Rezabakhsh^1,2,3^, Naser Aslanabadi^1^, Milad Asadi^4^, Venus Zafari^5^, Dariush Shanebandi^4^, Habib Zarredar^5^, Elgar Enamzadeh^1^, Hamed Taghizadeh^1^, Reza Badalzadeh^6,7^

1 Cardiovascular Research Center, Tabriz University of Medical Sciences, Tabriz, Iran, 2 Hematology, Immune Cell Therapy, and Stem Cells Transplantation Research Center, Clinical Research Institute, Urmia University of Medical Sciences, Urmia, Iran, 3 Emergency and Trauma Care Research Center, Tabriz University of Medical Sciences, Tabriz, Iran, 4 Immunology Research Center, Tabriz University of Medical Sciences, Tabriz, Iran, 5 Tuberculosis and Lung Diseases Research Center, Tabriz University of Medical Sciences, Tabriz, Iran, 6 Molecular Medicine Research Center, Tabriz University of Medical Sciences, Tabriz, Iran, 7 Department of Physiology, Faculty of Medicine, Tabriz University of Medical Sciences, Tabriz, Iran.
