# Supplementary figures and images for: Novel diagnostic potential of miR-1 in patients with acute heart failure
Source: PLoS One. 2022 Sep 23;17(9):e0275019. doi: 10.1371/journal.pone.0275019 (PMC9506628; doi:10.1371/journal.pone.0275019)

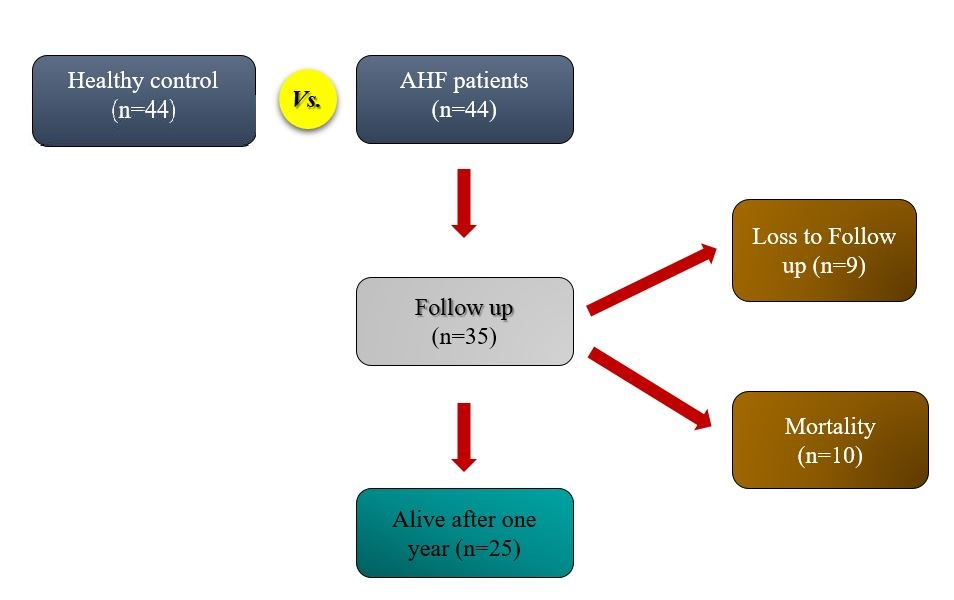

Supplement: S1 Fig — (TIF) [file pone.0275019.s001.tif]

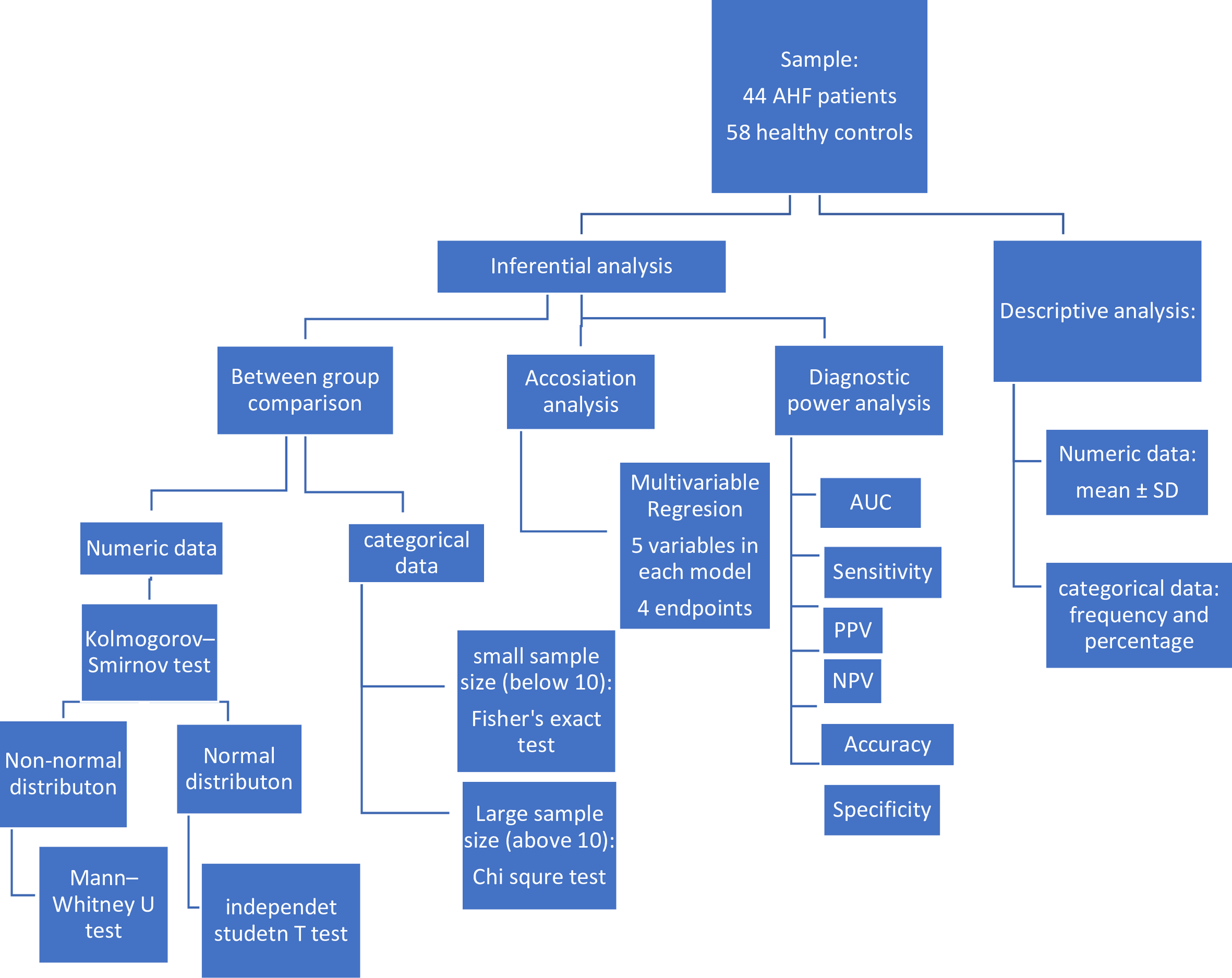

Supplement: S2 Fig — (TIF) [file pone.0275019.s002.tif]
