# Supplementary material for: Novel diagnostic potential of miR-1 in patients with acute heart failure
Source: PLoS One. 2022 Sep 23;17(9):e0275019. doi: 10.1371/journal.pone.0275019 (PMC9506628; doi:10.1371/journal.pone.0275019)
Supplement: S1 Table — (DOCX) [file pone.0275019.s003.docx]

| **miRNA** | **Optimal cut-point value^*^** | **Group**  **(n)** | | **AUC**  **(95% CI)** | **NRI**  **(95% CI)** | **Accuracy**  **(95% CI)** |
| --- | --- | --- | --- | --- | --- | --- |
|  |  | AHF | Healthy control |  |  |  |
| **miR-1** | ≥ 1.22 | 34 | 1 | 0.841  (0.75 to 94) | 0.35  (0.25 to 0.46) | 87.5  (78.7 to 93.5) |
|  | < 1.22 | 10 | 43 |  |  |  |
| **miR-21** | ≥1.12 | 31 | 6 | 0.857  (0.78 to 0.93) | 0.27  (0.16 to 0.37) | 78.4  68.3 to 86.4) |
|  | < 1.12 | 13 | 38 |  |  |  |
| **miR-23** | ≥1.24 | 31 | 1 | 0.837  (0.74 to 0.92). | 0.32  (0.21 to 0.42) | 84.0  (74.7 to 91.0) |
|  | < 1.24 | 13 | 43 |  |  |  |
| **miR-423** | ≥ 1.35 | 32 | 3 | 0.881  (0.80 to 0.95) | 0.31  (0.21 to 0.41) | 82.9  (73.4 to 90.1) |
|  | < 1.35 | 12 | 41 |  |  |  |

**Supplementary Table 1.** The performance comparison and validation of models for each miRNA for diagnosis of acute heart failure

AUC, area under the receiver operating characteristic (ROC) curve; NRI, net reclassification improvement; IDI, integrated discrimination improvement; miR or miRNA, micro ribonucleic acid; AHF, acute heart failure; 95% CI, 95% confidence interval
