# Supplementary material for: Novel diagnostic potential of miR-1 in patients with acute heart failure
Source: PLoS One. 2022 Sep 23;17(9):e0275019. doi: 10.1371/journal.pone.0275019 (PMC9506628; doi:10.1371/journal.pone.0275019)
Supplement: S2 Table — (DOCX) [file pone.0275019.s004.docx]

**Supplementary Table 2.** The level of miRNAs in patients who developed/not developed the adverse events during follow up

|  |  | | **n** | **mir1**  **Mean ± SD** | **mir21**  **Mean ±SD** | **mir23**  **Mean ± SD** | **mir423-5-p**  **Mean ± SD** |
| --- | --- | --- | --- | --- | --- | --- | --- |
| **Mortality data in the initial admission**  **(n = 44)** | **In hospital mortality** | Absent | 41 | 1.818 ± 0.819 | 1.474 ± 0.571 | 1.462 ± 0.589 | 1.728 ± 0.589 |
|  |  | Present | 3 | 1.940 ± 0.937 | 1.213 ± 0.240 | 1.533 ± 0.387 | 1.837 ± 0.150 |
|  | P value* | | | 0.759 | 0.405 | 0.571 | 0.436 |
| **Mortality data among those who were successfully followed for one year (n = 35)** | **Mortality in one year (including in-hospital mortality)** | Dead | 10 | 2.082 ± 0.920 | 1.350 ± 0.282 | 1.429 ± 0.407 | 1.742 ± 0.344 |
|  |  | Alive | 25 | 2.028 ± 0.855 | 1.415 ± 0.570 | 1.526 ± 0.579 | 1.646 ± 0.586 |
|  | P value* | | | 0.957 | 0.733 | 0.733 | 0.186 |
| **Among those alive after one year**  **(n = 25)** | **Readmission in one year** | Absent | 9 | 1.957 ± 0.656 | 1.643 ± 0.782 | 1.682 ± 0.821 | 1.970 ± 0.740 |
|  |  | Present | 16 | 2.068 ± 0.967 | 1.287 ± 0.381 | 1.438 ± 0.393 | 1.463 ± 0.399 |
|  | P value* | | | 0.598 | 0.187 | 0.598 | 0.065 |
|  | **FC after one year** | I | 11 | 1.993 ± 0.848 | 1.595 ± 0.689 | 1.638 ± 0.746 | 1.859 ± 0.740 |
|  |  | II | 6 | 2.150 ± 0.702 | 1.100 ± 0.547 | 1.253 ± 0.521 | 1.613 ± 0.524 |
|  |  | III | 3 | 1.254 ± 01.047 | 1.273 ± 0.323 | 1.533 ± 0.237 | 1.263 ± 0.249 |
|  |  | IV | 5 | 2.422 ± 0.869 | 1.482 ± 0.279 | 1.600 ± 0.321 | 1.444 ± 0.183 |
|  | P value# | | | 0.335 | 0.352 | 0.699 | 0.288 |

* Mann-Whitney U test, # Kruskal-Wallis test, ICD, FC, New York Heart Association (NYHA) Functional Classification; SD, standard deviation.
